# Applied Uses of Extracorporeal Membrane Oxygenation Therapy

**DOI:** 10.7759/cureus.5163

**Published:** 2019-07-17

**Authors:** Avani R Patel, Amar R Patel, Shivank Singh, Shantanu Singh, Imran Khawaja

**Affiliations:** 1 Internal Medicine, Northern California Kaiser Permanente, Fremont, USA; 2 Internal Medicine, Southern Medical University, Guangzhou, CHN; 3 Pulmonary Medicine, Marshall University School of Medicine, Huntington, USA

**Keywords:** extracorporeal membrane oxygenation (ecmo), venoarterial extracorporeal membrane oxygenation (va-ecmo), cardiogenic shock, respiratory failure, sepsis-induced cardiomyopathy, venovenous extracorporeal membrane oxygenation (vv-ecmo), pulmonary embolism, cardiopulmonary resuscitation (cpr), femoral vein, lung rest

## Abstract

Extracorporeal membrane oxygenation (ECMO) therapy has been around since the 1970s and has completely changed how critical care physicians view supportive therapy for certain patients. ECMO therapy is a supportive therapy provided by a mechanical extracorporeal circuit that is able to directly oxygenate and remove carbon dioxide from the blood. By performing this, ECMO can provide cardiac, respiratory, or combined cardiopulmonary supportive therapy in cases of failure. ECMO therapy also places less emphasis on invasive mechanical ventilation, which prevents barotrauma and gives rest to the lungs. Therefore, they are used for several different conditions. This review article focuses on the definition, principles, types, and practical applications of ECMO therapy.

## Introduction and background

Extracorporeal membrane oxygenation (ECMO) therapy has changed the way physicians view supportive treatment for cardiac, respiratory, or combined cardiopulmonary failure. ECMO therapy provides days to weeks of supportive treatment to patients with cardiac, respiratory, and combined cardiopulmonary failure [1]. ECMO was first reported in 1974 [1, 2]. From 2006 to 2011, the use of ECMO in adult patients has increased by more than 433% within the United States alone [1, 2]. The typical ECMO circuit consists of a blood pump, the membrane oxygenator (acts as an artificial lung), conduit tubing, a heat exchanger, as well as a drainage cannula and a return cannula [1]. It is a process that involves an extracorporeal circuit that directly oxygenates and removes carbon dioxide from the blood using an oxygenator. An oxygenator is a gas exchange device that uses a semipermeable membrane in order to separate a blood compartment from a gas compartment. The ECMO therapy process consists of deoxygenated blood withdrawn through a drainage cannula by an external pump, which then passes through the oxygenator, and is finally returned to the patient through a reinfusion cannula [3]. Briefly, the ECMO circuit drains blood from the venous system and after pumping it through the membrane oxygenator, the newly oxygenated blood is returned to the patient. By doing this, ECMO allows for a reduction of ventilator settings to decrease the chances of lung injury [1]. ECMO provides “lung rest,” by allowing lower tidal volumes and pressures. Therefore as proven in earlier studies, ECMO should be associated with a lower mortality rate because it allows for lower fractions of inspired oxygen to be used in mechanical ventilation [4]. Despite this, the data are still contradicting regarding the lower mortality rate in ECMO. The most recent randomized trial for ECMO in severe acute respiratory distress syndrome (ARDS) (ECMO to Rescue Lung Injury in Severe ARDS [EOLIA]) failed to show mortality benefit from using early ECMO despite benefit in secondary outcomes and possibly mortality when accounting for cross-over [5]. Also, if a mortality benefit is present, lower fractional concentrations of O_2_ in inspired gas (FiO_2_) may be one of the contributing factors but a cause-effect relationship with mortality is not certain. Nonetheless, ECMO therapy is highly utilized [5]. This review article examines the different applications of ECMO therapy in the intensive care unit setting. Our aim is to highlight the data that are present and bring notice to where data are lacking and could potentially benefit from further academic studies. Our aim is to highlight the present data and their limitations, as well as how these data could potentially benefit from further academic studies. 

## Review

Different modes of ECMO therapy

ECMO therapy subdivided into venovenous extracorporeal membrane oxygenation (VV-ECMO) and venoarterial extracorporeal membrane oxygenation (VA-ECMO).

VV-ECMO is a process where the blood is drained from a central vein and returned to another central vein with the device only providing gas exchange [4]. By utilizing this bridging therapy, oxygen support is provided while the lungs recover in respiratory failure [3]. VV-ECMO involves venous cannulation at two different sites for drainage and reinfusion of the blood [6]. A VV-ECMO normally has two access sites so it requires one femoral venous access for drainage and one femoral venous access for reinfusion. With the addition of the bicaval dual-lumen cannula, the internal jugular vein can be used as a lone access site for VV-ECMO [3, 7-8]. 

VA-ECMO is a process where the blood is drained from the venous system and pumped into an artery with the circuit providing both respiratory and circulatory support [4]. This is done for patients who have compromised cardiac function with or without impaired gas exchange. The VA-ECMO circuit typically consists of femoral vein drainage and reinfusion through the femoral artery [3].

Uses in cardiac failure

Cardiogenic Shock for Non-myocardial Infarction Etiologies

The definition of cardiogenic shock is a primary cardiac disorder that results in both clinical and biochemical evidence of tissue hypoperfusion. Cardiogenic shock is clinically diagnosed by a systolic blood pressure (BP) of ≤ 90 mm Hg either with or without support, for 30 or more minutes, and a urine output ≤ 30 mL/hour or cool extremities [9]. Cardiogenic shock hemodynamic criteria will have a decreased cardiac index (≤ 2.2 L/min/m^2^ body surface area) and an increased pulmonary-capillary wedge pressure (> 15 mm Hg) [9]. 

One application of ECMO is supportive treatment for non-ischemic cardiogenic shock, including cardiogenic shock caused by fulminant myocarditis [10-12]. The World Health Organization and the International Society and Federation of Cardiology defined myocarditis as an inflammatory disease of the myocardium, which is diagnosed by established histological, immunological, and immunohistochemical criteria [13]. As seen with previous prospective studies, fulminant myocarditis that is treated with percutaneous ECMO has been proven to have a good clinical outcome that is comparable to outcomes seen in patients of non-fulminant myocarditis [10]. 

Cardiogenic Shock Complicating Acute Myocardial Infarction

Cardiogenic shock secondary to acute myocardial infarction is also treated with VA-ECMO therapy. It offers an advantage over traditional medical therapy (inotropes and vasopressors) in that cardiac output is increased without putting an increased demand on myocardial tissue. It also offers the benefit of rapid insertion, biventricular support, and lung rest in cases of simultaneous respiratory failure [3]. Early ECMO therapy initiation in patients with cardiogenic shock secondary to myocardial infarction has demonstrated improved 30-day outcomes in patients [14]. In a different study performed two years later, it was confirmed that patients benefited with improved 30-day outcomes and that their one-year outcome was also better with the ECMO therapy [15].

Sepsis-associated Cardiomyopathy

Septic shock can lead to cardiac and hemodynamic failure. In those cases, VA-ECMO can be used as supportive therapy. Previous studies have determined that pediatric patients with refractory septic shock have been associated with good clinical outcomes after VA-ECMO but there is a lack of consensus for adult patients [16]. Some studies have determined that adult patients of septic shock improve greatly after VA-ECMO therapy [16-17]. One retrospective, single-center, observational study and a cross-sectional survey determined that VA-ECMO therapy rescued more than 70% of patients who developed refractory cardiovascular dysfunction secondary to severe bacterial septic shock with survivors also reporting good long-term quality of life [16]. Another prospective study found that the results of VA-ECMO therapy in septic shock patients were unsatisfactory. In addition, their study also determined that ECMO therapy might be contraindicated in patients aged 60 years and older [18]. Therefore, to reach a decision on the standard of care, more research needs to be done.

Pulmonary Embolism

ECMO therapy has also demonstrated better clinical outcomes with massive pulmonary embolism, which is fatal in patients because it can cause acute irreversible pulmonary and cardiac failure [19-20]. In 2007, a retrospective study was performed at the University of Michigan Medical Center for determining whether using ECMO therapy in massive pulmonary embolism patients improved their prognosis. The average duration of ECMO therapy utilization for survivors was 5.4 days, ranging from 5 hours to 12.5 days. The overall survival rate was 62% (13/21) when combined with anticoagulation or surgical embolectomy [19]. A prospective study conducted in 2012 similarly demonstrated an improved prognosis in 30-day survival rate of patients (70%) [20]. 

Post-operative Cardiogenic Shock and Post-transplant Primary Graft Failure

Post-operative cardiogenic shock refers to post-cardiotomy cardiogenic shock, which is a rare and deadly complication of cardiac surgery [3]. A 2012 study with 517 patients determined that ECMO therapy given to post-cardiotomy cardiogenic shock patients still leads to a high morbidity and high mortality rate. Therefore, it becomes crucial to administer the treatment in patients based on their individual risk profiles rather than as a standard of care [21]. 

ECMO therapy is also used in primary graft failure secondary to heart transplantation, which is also associated with a high mortality [22-23]. In a previous retrospective study, ECMO-supported patients showed a good survival-to-discharge rate [24].

Bridge to ventricular assist device (VAD) Implantation or Heart Transplantation

A VAD is an implantable mechanical pump that helps pump blood from the ventricles to the rest of the body. It is used in patients of cardiac failure and as a bridging therapy for patients awaiting heart transplantation [25].

ECMO therapy is used as a bridging therapy to heart transplantation or VAD implantation, or it can be used as supportive therapy to stabilize the patient till their prognosis becomes known [26-27]. In a previous prospective study, it was determined that ECMO is a valid method of supporting patients awaiting high-urgency heart transplantation and can be used as a short-term bridge to surgery [27]. Despite this, as physicians, we still need more studies to address the relative and absolute contraindications of mechanical circulatory support [3]. 

Prevention of Right Ventricular Failure After LVAD Implantation

Once a patient has an implanted VAD, then it becomes important to allow time for the already compromised right ventricle to adjust to the increasing preload. By using ECMO therapy, right ventricular distention and failure can be avoided [3]. Studies performed in 2011 and 2012 have demonstrated that ECMO is an excellent supportive therapy for patients with recently implanted VAD. Improved patient prognosis was documented [28-29].

Pulmonary Hypertension

VA-ECMO therapy has become an important part of therapy for pulmonary hypertension complicated further by right ventricular failure, suboptimal medical therapy, or future lung transplantation [30]. During ECMO therapy, pulmonary vasodilators may be optimized for maximizing treatment effectiveness [30]. Pulmonary hypertension patients have high pulmonary vascular resistance (≥ 25 mm Hg), which can lead to right heart failure and eventually death without proper treatment [3]. Therefore, it becomes important to provide ECMO cannulation that can deliver the appropriate therapy, bypass the pulmonary vasculature, and decompress the right ventricle [3]. Femoral cannulation is avoided [3]. Studies have discovered that one option proven to work is having a drainage cannula in the internal jugular vein with a reinfusion cannula inserted into the subclavian artery [31]. An arteriovenous ECMO can be inserted between the main pulmonary artery and the left atrium after a sternotomy [32]. An oxygenated right-to-left shunt can be created for patients with pre-existing atrial defects while also decompressing the right ventricle [33-34].

Uses in respiratory failure

Acute Respiratory Distress Syndrome

ARDS is one of the most studied indications for ECMO therapy. ARDS is a life-threatening condition caused by capillary endothelial injury and diffuse alveolar damage, and leads to poor oxygenation and non-compliant lungs [35]. Volume and pressure-limited ventilation strategy is the only ventilation strategy proven to reduce ARDS mortality because positive-pressure ventilation may cause barotrauma [36-37]. Low-tidal volume can also cause further problems in ARDS patients like hypercapnia and respiratory acidosis. ECMO can help give the lungs rest and correct these problems [38]. Several studies have demonstrated the benefit of ECMO therapy in ARDS. One was the CESAR trial which determined that an ECMO-based management protocol would significantly improve survival without severe disability [39]. The CESAR trial was a multicentre randomized clinical trial (RCT). In contrast, the EOLIA 2018 trial (another RCT) determined that ARDS 60-day mortality was not significantly lower with ECMO therapy [5].

Hypercapnic Respiratory Failure

Hypercapnic respiratory failure (HRF) is defined as chronic respiratory failure characterized by elevated levels of carbon dioxide in the blood. HRF is mainly seen as a result of chronic obstructive pulmonary disease or long-standing obesity hypoventilation syndrome. Giving the patient treatment with invasive mechanical ventilation will lead to complications like dynamic hyperinflation and elevations in intrinsic positive end-expiratory pressure, ventilator-associated pneumonia, and impaired delivery of aerosolized medications [40-41]. Because of these potential complications, ECMO therapy has been found to be an excellent supportive therapy for HRF [3]. This has been confirmed with clinical trials, where ECMO therapy improved the prognosis of HRF patients [42].

Bridge to Lung Transplantation and Post-transplantation Primary Graft Dysfunction

ECMO therapy has been proven to provide appropriate (pulmonary and/or circulatory) support for patients waiting for lung transplantation [3]. Many transplantation surgeries develop primary graft failure, which is the first cause of early mortality after lung, and heart-lung transplantation [3]. The International Society for Heart-Lung Transplantation Registry determined a 24.7% and 27% mortality rate for lung and heart-lung transplantation, respectively. The underlying cause was primary graft dysfunction during the first 30 days after the operation [43]. Because of this, mechanical support therapy such as VA-ECMO is utilized. It has been determined that VA-ECMO can improve survival rates (82%) of primary graft rejection patients [44-46]. 

Use in cardiopulmonary resuscitation

ECMO therapy has also been applied to cardiopulmonary resuscitation (CPR). In fact, studies performed on the effectiveness of ECMO plus CPR show improved short-term and long-term benefits compared to conventional CPR [47-48]. One 2008 study showed a significant difference in survival-to-discharge rate (hazard ratio [HR] 0.51, 95% confidence interval [CI] 0.35-0.74, p-value<0.0001), 30-day survival (HR 0.47, 95% CI 0.28-0.77, p-value=0.003), and one-year survival (HR 0.53, 95% CI 0.33-0.83, p-value=0.006) favoring ECMO therapy with CPR over conventional CPR [47].

A second study in 2013 determined that the extracorporeal membrane oxygenation with cardiopulmonary resuscitation (E-CPR) patient group had better two-year survival with minimal neurological impairment than the conventional cardiopulmonary resuscitation (C-CPR) patient group [48]. The two-year survival with minimal neurological impairment was fourfold higher in the E-CPR group than that in the C-CPR group (20.0% versus 5.0%, HR=0.53, 95% CI=0.36-0.80, p-value=0.002) [48]. 

## Conclusions

With the many uses of ECMO therapy, it becomes important for physicians both in and out of the hospital to familiarize themselves with the subject. This is a review article for those physicians regarding the background, the principle, the types, and the uses of ECMO therapy in different types of organ failure. Despite our current breadth of knowledge, more studies need to be done. We still need more data on ECMO therapy contraindications and complications, as well as its uses in high mortality conditions. Our hope is that more physicians will strive for larger prospective studies in order to address these unanswered questions.
